# Efficacy of 5-aminolevulinic acid photodynamic therapy for the treatment of female lower reproductive tract intraepithelial lesions and its predictive biomarker: DNA methylation

**DOI:** 10.3389/fmed.2026.1828763

**Published:** 2026-06-25

**Authors:** Qin Han, Zhangxin Wu, Hongyan Guo

**Affiliations:** Department of Gynecology, Peking University Third Hospital, Haidian, China

**Keywords:** HSIL, JAM3, PAX1, photodynamic therapy, VaIN

## Abstract

**Objective:**

This prospective study aimed to evaluate the efficacy of 5-aminolevulinic acid-mediated photodynamic therapy (ALA-PDT) for cervical/vaginal intraepithelial neoplasia (CIN/VaIN) and explore the predictive value of pretreatment PAX1/JAM3 methylation status in exfoliated cells for treatment outcomes.

**Methods:**

A total of 516 patients with histologically confirmed high-grade squamous intraepithelial lesion (HSIL: CIN2/3, VaIN2/3) who received ALA-PDT between September 2024 and February 2026 were enrolled. Pretreatment exfoliated cells were collected for HR-HPV detection, ThinPrep cytology test (TCT), and PAX1/JAM3 methylation analysis via multiplex real-time quantitative PCR. All patients received two courses of ALA-PDT consisting of six treatment sessions in total at 7–14-day intervals. Each session was performed with 20% ALA topical application for 3 h followed by irradiation at 80 mW/cm^2^, 635 nm, for 25–30 min. Efficacy was evaluated by pathological regression, HR-HPV clearance at 6/12 months, and factors influencing outcomes were analyzed using univariate/multivariate regression and ROC curves.

**Results:**

The overall disease remission rates were 92.21% (CIN2), 88.68% (CIN3), 78.57% (VaIN2), and 66.67% (VaIN3). The HR-HPV clearance rates at 6 months were 60.79% for CIN2, 61.90% for CIN3, 52.38% for VaIN2, and 50.00% for VaIN3; the corresponding 12-month clearance rates were 71.72, 74.36, 58.54, and 50.00%, respectively. Univariate analysis identified prior cervical surgery, HPV16/18 positivity, TCT ≥ LSIL, and multiple lesions as adverse factors for CIN2. TCT ≥ LSIL, and multiple lesions were associated with poorer outcomes in VaIN2/3. PAX1/JAM3 hypomethylation was significantly associated with higher remission rates (*P* < 0.001). Pretreatment PAX1/JAM3 hypermethylation showed predictive value for incomplete response to ALA-PDT. Combined methylation detection showed optimal predictive performance (AUC: 0.861 for CIN2, 0.734 for CIN3, 0.777 for VaIN2, 0.750 for VaIN3).

**Conclusion:**

ALA-PDT is effective for HSIL with favorable HPV clearance. Pretreatment PAX1/JAM3 methylation status serves as a reliable non-invasive biomarker for predicting PDT efficacy, supporting individualized clinical decision-making.

## Background

Cervical cancer is the fourth most common malignant tumor among women worldwide and the fourth leading cause of cancer-related death in females globally. Each year, there are approximately 604,000 new cases and 342,000 deaths attributed to cervical cancer worldwide (1). In China, around 109,000 new cases and 59,000 deaths occur annually, accounting for roughly one-fifth of the global disease-related mortality (2).

Persistent infection with high-risk human papillomavirus (HR-HPV) is the primary etiological factor for cervical cancer and precancerous lesions (3). Approximately 80% of women will be infected with HPV at least once in their lifetime, and persistent HR-HPV infection may progress to high-grade cervical intraepithelial neoplasia (CIN2/3) and eventually develop into cervical cancer (4, 5).

Current therapeutic strategies for high-grade intraepithelial lesions of the female lower reproductive tract mainly include surgical resection and physical therapy. Cervical conization may disrupt cervical anatomy, potentially increasing the risk of miscarriage and preterm birth. Vaginal intraepithelial lesions often exhibit extensive distribution and special histological characteristics, making complete surgical excision technically challenging and anatomical reconstruction difficult. Traditional physical therapies, such as cryotherapy and CO_2_ laser ablation, achieve an efficacy rate of approximately 60–80% (6). However, suboptimal outcomes may occur in patients with multiple lesions, folded lesions, or lesions that cannot be fully exposed or flattened. Additionally, adjacent normal tissues may sustain varying degrees of damage during these procedures.

Photodynamic therapy (PDT) is a targeted physical treatment modality. A photosensitizer is selectively absorbed by metabolically active diseased cells and metabolized into cytotoxic reactive oxygen species (ROS) upon excitation by light of a specific wavelength, inducing apoptosis and necrosis of pathological cells without damaging extracellular matrix components such as fibrous connective tissue (7, 8). Consequently, PDT has the advantages of high selectivity, limited tissue destruction, and favorable wound healing. Currently, 5-aminolevulinic acid (ALA) is one of the most commonly used photosensitizers in clinical practice. Because the female lower reproductive tract is anatomically accessible for topical drug application and light exposure, ALA-PDT has been increasingly explored in gynecological diseases. Previous studies have reported its application in persistent HPV infection, low-grade cervical intraepithelial lesions, and cervical intraepithelial neoplasia (9–11). Our research team and others have also reported the safety and efficacy of PDT in treating cervical and vaginal precancerous lesions (12–14). However, clinical responses to PDT vary considerably among individuals. Some patients experience residual lesions or recurrence after treatment, which may necessitate retreatment or even surgical intervention. Previous studies on factors influencing PDT efficacy has primarily focused on HPV infection status, ThinPrep cytology test (TCT) results, and colposcopic transformation zones, but the conclusions remain inconsistent. Therefore, identifying reliable biomarkers for predicting PDT response is crucial for selecting suitable candidates, optimizing therapeutic regimens, and improving patient prognosis.

DNA methylation, a key component of epigenetics, refers to the addition of a methyl group to the 5-position carbon atom of cytosine in DNA molecules under the catalysis of DNA methyltransferases. Aberrant DNA methylation is closely associated with the initiation and progression of tumors and precancerous lesions. Previous studies have demonstrated that methylation abnormalities in host-cell genes are involved in cervical carcinogenesis and may serve as useful molecular markers for cervical lesion screening and risk stratification (15, 16). Hypermethylation of the paired box gene 1 (PAX1) and junctional adhesion molecule 3 (JAM3) (PAX1m and JAM3m) is widely investigated for the detection of CIN3+ lesions and could reduce unnecessary colposcopic referrals compared with cytology-based triage (17, 18). The methylation status of these genes may reflect biological characteristics related to cell proliferation, apoptosis, and treatment sensitivity. Since cervical and vaginal exfoliated cells can be collected non-invasively before treatment, detection of PAX1/JAM3 methylation status may provide a convenient approach for predicting the therapeutic efficacy of PDT.

Based on this, the present prospective study aimed to evaluate the efficacy of PDT in treating cervical and vaginal precancerous lesions and investigate the correlation between pretreatment PAX1/JAM3 methylation status in exfoliated cells and PDT outcomes, thereby providing a reference for individualized clinical decision-making.

## Study methods

### Study population

Between September 2024 and February 2026, patients who visited the Gynecology Clinic of Peking University Third Hospital and were diagnosed with cervical/vaginal intraepithelial neoplasia via colposcopy and pathological biopsy were enrolled. Following consultation with clinicians, patients who opted for ALA-PDT were included in the study. This was a prospective cohort study. All participants signed an informed consent form for ALA-PDT prior to treatment.

#### Inclusion criteria

(1) Age: 18–70 year-old; (2) Histologically confirmed cervical/vaginal high-grade squamous intraepithelial lesion (HSIL) via colposcopy and biopsy (including cervical CIN2 or CIN3, and/or vaginal VaIN2 or VaIN3); (3) Adequate colposcopic visualization with analyzable images; (4) Endocervical curettage (ECC) not indicating higher-grade lesions; (5) Every CIN3 patients did enhanced MR and report must show no suspicious infiltrative lesions.

#### Exclusion criteria

(1) Concurrent or suspected malignancy; (2) Porphyria or known hypersensitivity to red/blue light; (3) Uncontrolled severe comorbidities (e.g., abnormal liver/kidney function, coagulation disorders, heart failure, acute asthma exacerbation); (4) Systemic acute infection or unstable clinical status.

During treatment, clinical data were collected, including age, HPV infection type, TCT results, lesion distribution characteristics, cervical transformation zone type, vaginal microecology, reproductive history, and surgical history.

This study was approved by the Bioethics Committee at the Peking University Third Hospital (IRB00006761-M2024575). The study protocol was reviewed and approved before patient enrollment, and written informed consent was obtained from all participants.

### HR-HPV detection, cytological examination, and colposcopy-guided pathological biopsy

For all participants, sample collection was performed before colposcopy, acetic acid/iodine application, and biopsy. Samples for cytology and HR-HPV testing were collected first, followed by sample collection for PAX1/JAM3 methylation testing using a separate sampling brush.

A cytobrush was used to collect vaginal and cervical secretions. HR-HPV detection was performed using the Cobas^®^ HPV test (Roche Molecular Diagnostics, Basel, Switzerland) according to the manufacturer’s instructions, which targets 14 high-risk HPV genotypes (HPV 16, 18, 31, 33, 35, 39, 45, 51, 52, 56, 58, 59, 66, and 68). Pap smear samples were collected in ThinPrep fixative solution, processed, stained, and analyzed using an automated stainer. Colposcopy was performed in accordance with updated cervical cancer screening guidelines using a light-emitting diode (LED) colposcope at a focal distance of 300 mm. The entire abnormal epithelial area was inspected following application of 3% acetic acid and subsequent iodine solution (essential for assessing vaginal neoplasia and determining lesion extent). Suspicious lesions were identified as iodine-negative (non-stained) areas, where biopsy specimens were obtained for pathological examination. Colposcopy was repeated post-treatment.

### DNA methylation detection

All patients underwent pretreatment DNA methylation testing immediately before colposcopy. Pretreatment PAX1/JAM3 methylation status was used for the primary predictive analysis of PDT efficacy. During post-treatment follow-up, DNA methylation testing was recommended at each scheduled follow-up visit and was performed when clinically indicated or when the patient agreed. Follow-up methylation results were not used to define the primary efficacy endpoint. Samples for methylation testing were collected after cytology and HR-HPV samples had been obtained. Exfoliated cells were collected from the cervical/vaginal surface and cervical canal using a specialized plastic brush rotated clockwise five times, and samples were stored in vials containing cell-fixative solution labeled with unique detection barcodes. DNA methylation analysis was performed within 1 week of sampling using a commercial detection kit (Beijing OriginPoly Bio Tec Co., Ltd.). Briefly:

1. Genomic DNA was extracted from exfoliated cervical cells using the JH-DNA separation and purification kit (Spin Column), and DNA concentration and quality were quantified.
2. DNA samples were subjected to bisulfite conversion.
3. Methylation status of target genes (PAX1 and JAM3) was detected using multiplex real-time quantitative PCR (SLAN-96S Automatic Medical PCR Analysis System), with GAPDH as the internal reference gene.

#### PCR reaction conditions

Pre-denaturation at 96°C for 10 min; 45 cycles of denaturation at 94°C for 15 s, annealing at 64°C for 5 s, and extension with fluorescence acquisition at 60°C for 30 s; final cooling at 25°C for 1 min. After amplification, Ct values for JAM3 (ΔCtJ), PAX1 (ΔCtP), and the internal reference gene were calculated as ΔCt = Ct (target gene) - Ct (internal reference gene). A smaller ΔCt value indicated a higher methylation level. The cutoff values for hypermethylation (high risk) were defined as ΔCtP ≤ 6.6 (PAX1) and ΔCtJ ≤ 10.0 (JAM3).

### ALA-PDT protocol

Following the Chinese expert consensus on the clinical applications of aminolevulinic acid-based photodynamic therapy in female lower genital tract diseases (2022) (19), ALA-PDT treatment was performed according to the following standardized protocol.

Unprepared ALA was stored in the dark. A 20% ALA dispersion (Shanghai Fudan Zhangjiang Bio-Pharmaceutical Co., Ltd., Shanghai, China) was freshly prepared in normal saline at a single dose of 38 mg/cm^2^. Treatment was performed using a light-emitting diode (LED)-based photodynamic therapy device (LED-IB, Wuhan Yage Optic and Electronic Technique Co., Ltd., Wuhan, China). Topical application of ALA was maintained for 3 h, followed by light irradiation (wavelength: 635 nm) at a power density of 80 mW/cm^2^ for 25–30 min at a focal distance of 5 cm. ALA-PDT was administered every 7–14 days, avoiding the menstrual period, and all patients received two treatment courses consisting of six sessions in total.

### Efficacy evaluation and follow-up

Colposcopy was performed 4 weeks post-treatment to assess initial response. All patients were followed up at 6 and 12 months post-treatment, with efficacy evaluated by HPV genotyping, TCT, and colposcopy-directed biopsy if clinically indicated per guidelines. At each scheduled follow-up visit, HPV genotyping and TCT were routinely performed, and DNA methylation testing was recommended as described above. Annual follow-up was recommended for all participants.

#### Primary endpoint

Pathological regression post-treatment. Patients were categorized as:

- *Disease remission:* Repeat biopsy confirming low-grade squamous intraepithelial lesion (LSIL) or no evidence of squamous intraepithelial lesion (SIL).
- *Persistent disease:* Lesions of the same histological grade.
- *Progressive disease:* Lesions of higher histological grade.

HR-HPV clearance rates at 6 and 12 months post-treatment were also evaluated as secondary endpoints.

### Statistical analysis

Statistical analyses were performed using IBM SPSS Statistics version 24.0. Categorical variables were presented as numbers and percentages, and continuous variables were expressed as medians with interquartile ranges. Categorical variables were compared using the chi-square test or Fisher’s exact test, and continuous variables were compared using the Mann–Whitney U test. Univariate and multivariate regression analyses were performed to identify factors associated with treatment outcomes after PDT. Receiver operating characteristic (ROC) curves were constructed to analyze biomarkers predicting histological regression, with sensitivity, specificity, area under the curve (AUC) and 95% confidence intervals (Cl) calculated using exact binomial methods. For ROC analysis, persistent disease was coded as “1,” whereas disease remission was coded as “0.” Pretreatment PAX1/JAM3 hypermethylation was treated as a positive test result for predicting incomplete response to PDT. The methylation status was classified according to the prespecified cutoffs (20) Correlations between molecules were assessed using the Pearson product-moment correlation coefficient. All analyses were based on the full-analysis set, with a two-sided *P* < 0.05 considered statistically significant.

## Results

### Population characteristics

A total of 516 patients with histologically confirmed CIN/VaIN who received PDT for female genital tract intraepithelial neoplasia at the Gynecology Outpatient Clinic of Peking University Third Hospital between September 2024 and February 2026 were included. Demographic and clinical data, including HPV and TCT results, lesion distribution, smoking history, and prior uterine surgery history, were collected (Table 1). Other information was not included in the final regression analysis because of incomplete data, limited event numbers, avoiding model overfitting.

**TABLE 1:** Baseline characteristics of the study population.

Grade of lesion	CIN 2 (*N* = 398)	CIN 3 (*N* = 53)	VaIN 2 (*N* = 56)	VaIN 3 (*N* = 9)
Age	37 (23∼52)	30 (24∼33)	54 (39∼67)	57 (45∼63)
Menopause	4 (1.01%)	0 (0.00%)	39 (69.64%)	7 (77.78%)
Smoking	9 (2.26%)	1 (1.89%)	2 (3.57%)	1 (11.11%)
History of cervical surgery				
LEEP history	37 (9.30%)	0 (0.00%)	2 (3.57%)	0 (0.00%)
CKC history	19 (4.77%)	0 (0.00%)	4 (7.14%)	1 (11.11%)
Hysterectomy history	0 (0.00%)	0 (0.00%)	29 (51.79%)	5 (55.56%)
HPV				
Negative	17 (4.27%)	0 (0.00%)	1 (1.79%)	0 (0.00%)
HPV16 ± other high-risk HPV positive	122 (30.65%)	18 (33.96%)	12 (21.43%)	4 (44.44%)
HPV18 ± other high-risk HPV positive	83 (20.85%)	4 (7.55%)	7 (12.50%)	1 (11.11%)
HPV16/18 both ± other high-risk HPV positive	21 (5.28%)	3 (5.36%)	2 (3.57%)	1 (11.11%)
Other high-risk HPV positive	155 (38.94%)	28 (52.83%)	34 (60.71%)	3 (33.34%)
TCT				
NILM	127 (31.91%)	3 (5.66%)	29 (51.79%)	2 (22.22%)
ASC-US	103 (25.88%)	4 (7.55%)	17 (30.36%)	3 (33.34%)
ASC-H	55 (13.82%)	9 (16.98%)	0 (0.00%)	1 (11.10%)
LSIL	113 (28.39%)	33 (62.26%)	10 (17.86%)	3 (33.34%)
HSIL	0 (0.00%)	4 (7.55%)	0 (0.00%)	0 (0.00%)
DNA methylation (before treatment)				
PAX1 (hypo)	340 (85.42%)	31 (58.49%)	47 (83.93%)	7 (77.78%)
PAX1 (hyper)	58 (14.57%)	22 (41.51%)	9 (16.07%)	2 (22.22%)
JAM3 (hypo)	354 (88.94%)	21 (39.62%)	51 (91.07%)	6 (66.67%)
JAM3 (hyper)	44 (11.06%)	32 (60.38%)	5 (8.93%)	3 (33.33%)
Pathological characteristics				
Single lesion of HSIL	179 (44.97%)	37 (69.81%)	42 (75.00%)	8 (88.89%)
Multiple lesions of HSIL	219 (55.03%)	16 (30.19%)	14 (25.00%)	1 (11.11%)
Glandular involvement	163 (40.95%)	2 (3.77%)	0 (0.00%)	0 (0.00%)

LEEP, Loop electrosurgical excision procedure; CKC, Cold knife conization; NILM, Negative for intraepithelial lesion or malignancy; ASC-US, Atypical squamous cells of undetermined significance; ASC-H, Atypical squamous cells, cannot exclude HSIL; LSIL, Low-grade squamous intraepithelial lesion; HSIL, High-grade squamous intraepithelial lesion; PAX1, paired boxed 1 gene; JAM3, junctional adhesion molecule 3 gene; PAX1 (hypo), PAX1 hypomethylation; PAX1 (hyper), PAX1 hypermethylation; JAM3 (hypo), JAM3 hypomethylation; JAM3 (hyper), JAM3 hypermethylation.

### Therapeutic response

After completion of the scheduled PDT treatment courses, patients underwent colposcopic evaluation. For patients with persistent or progressive disease, further treatment options (additional PDT courses or alternative therapies) were discussed jointly by clinicians and patients. As shown in Table 2, the remission rates were 92.21% for CIN2, 88.68% for CIN3, 78.57% for VaIN2, and 66.67% for VaIN3. Among patients achieving disease remission, the overall HR-HPV clearance rate was 59.95% (265/442) at 6 months and 70.39% (302/429) at 12 months post-treatment.

**TABLE 2:** Therapeutic response of HSIL to ALA-PDT.

Grade of lesion	CIN 2 (*N* = 398)	CIN 3 (*N* = 53)	VaIN 2 (*N* = 56)	VaIN 3 (*N* = 9)
Therapeutic response				
Disease remission	367 (92.21%)	47 (88.68%)	44 (78.57%)	6 (66.67%)
Persistent disease	31 (7.79%)	6 (11.32%)	12 (21.43%)	3 (33.33%)
Progressive disease	0 (0.00%)	0 (0.00%)	0 (0.00%)	0 (0.00%)
Remission rates of HR-HPV				
6 months after treatment	60.79% (214/352)	61.90% (26/42)	52.38% (22/42)	50.00% (3/6)
12 months after treatment	71.72% (246/343)	74.36% (29/39)	58.54% (24/41)	50.00% (3/6)

### Factors influencing treatment efficacy and predictive biomarkers

Univariate analysis identified several factors associated with PDT outcomes (Table 3):

**TABLE 3:** Univariate analysis of factors associated with disease remission after ALA-PDT.

Factors	Subgroups	CIN2, disease remission n/N (%)	*P*	CIN3, disease remission n/N (%)	*P*	VaIN2, disease remission n/N (%)	*P*	VaIN3, disease remission n/N (%)	*P*
Smoking	Yes	7/9 (77.78%)	0.102	0/1 (0.00%)	0.113	1/2 (50.00%)	0.343	0/1 (0.00%)	0.333
	No	360/389 (92.54%)		47/52 (90.38%)		43/54 (79.63%)		6/8 (75.00%)	
History of cervical/uterine treatment	Yes	28/56 (50.00%)	< 0.001	0/0 (NA)	NA	27/35 (77.14%)	1.000	4/6 (66.67%)	0.988
	No	339/342 (99.12%)		47/53 (88.68%)		17/21 (80.95%)		2/3 (66.67%)	
HPV	HPV16/18 ± other high-risk HPV positive	214/226 (94.69%)	0.034	22/25 (88.00%)	1.000	16/21 (76.19%)	0.748	3/6 (50.00%)	0.774
	Negative or other high-risk HPV positive	153/172 (88.95%)		25/28 (89.29%)		28/35 (80.00%)		3/3 (100.00%)	
TCT	≤ ASC-H	273/285 (95.79%)	< 0.001	14/16 (87.50%)	1.000	40/46 (86.96%)	0.046	4/6 (66.67%)	1.000
	≥ LSIL	94/113 (83.19%)		33/37 (89.19%)		4/10 (40.00%)		2/3 (66.67%)	
Lesions of HSIL	Single lesion	179/179 (100.00%)	< 0.001	34/37 (91.89%)	0.392	37/42 (88.10%)	0.026	6/8 (75.00%)	1.000
	Multiple lesions	188/219 (85.84%)		13/16 (81.25%)		7/14 (50.00%)		0/1 (0.00%)	

ALA-PDT, 5-aminolevulinic acid-mediated photodynamic therapy; CIN, cervical intraepithelial neoplasia; VaIN, vaginal intraepithelial neoplasia; HPV, human papillomavirus; TCT, ThinPrep cytology test; ASC-H, atypical squamous cells, cannot exclude high-grade squamous intraepithelial lesion; LSIL, low-grade squamous intraepithelial lesion; HSIL, high-grade squamous intraepithelial lesion.

1. For CIN2: Prior cervical treatment history, HPV16/18 positivity, TCT results ≥ LSIL, and multiple lesions were associated with poorer treatment outcomes.
2. For CIN3: No significant prognostic factors were identified.
3. For vaginal lesions (VaIN2/VaIN3): TCT results ≥ LSIL, and multiple lesions were associated with adverse outcomes.

The correlation between PAX1/JAM3 methylation levels and therapeutic outcomes was further analyzed (Table 4). For all lesion grades, hypomethylation of PAX1 and JAM3 was significantly associated with higher remission rates, while hypermethylation correlated with persistent disease. To evaluate the predictive value of methylation detection for the effectiveness of PDT, persistent disease was defined as “1” and disease remission was defined as “0” for the binary classification. Table 5 evaluates the predictive performance of pretreatment PAX1/JAM3 methylation status for post-treatment pathological response. AUC demonstrated that both genes exhibited favorable predictive value for PDT efficacy, with combined detection of PAX1 and JAM3 methylation further improving prediction accuracy (Table 5 and Supplementary Figure 1).

**TABLE 4:** PAX1 and JAM3 methylation status in different treatment outcome groups.

Methylation	PAX1 (hypo)	PAX1 (hyper)	JAM3 (hypo)	JAM3 (hyper)	PAX1 (hypo) and JAM3 (hypo)	PAX1 (hyper)/ JAM3 (hyper)	PAX1 (hypo)/ JAM3 (hypo)	PAX1 (hyper) and JAM3 (hyper)
CIN 2 (398)								
Disease remission (367)	90.46% (332/367)	9.54% (35/367)	94.82% (348/367)	5.17% (19/367)	88.28% (324/367)	10.08% (37/367)	95.37% (350/367)	4.63% (17/367)
Persistent disease (31)	25.81% (8/31)	74.19% (23/31)	19.35% (6/31)	80.65% (25/31)	16.13% (5/31)	87.10% (27/31)	32.26% (10/31)	67.74% (21/31)
CIN3 (53)								
Disease remission (47)	63.83% (30/47)	36.17% (17/47)	44.68% (21/47)	55.32% (26/47)	40.43% (19/47)	59.57% (28/47)	68.09% (32/47)	31.91% (15/47)
Persistent disease (6)	16.67% (1/6)	83.33% (5/6)	0.00% (0/6)	100.00% (6/6)	0.00% (0/6)	100.00% (6/6)	16.67% (1/6)	83.33% (5/6)
VaIN 2 (56)								
Disease remission (44)	95.45% (42/44)	4.55% (2/44)	97.73% (43/44)	2.27% (1/44)	88.64% (39/44)	4.55% (2/44)	97.73% (43/44)	2.27% (1/44)
Persistent disease (12)	41.67% (5/12)	58.33% (7/12)	66.67% (8/12)	41.67% (5/12)	33.33% (4/12)	66.67% (8/12)	66.67% (8/12)	33.33% (4/12)
VaIN 3 (9)								
Disease remission (6)	100.00% (6/6)	0.00% (0/6)	83.33% (5/6)	16.67% (1/6)	83.33% (5/6)	16.67% (1/6)	100.00% 0.00% (6/6)	0.00% (0/6)
Persistent disease (3)	33.33% (1/3)	66.67% (2/3)	33.33% (1/3)	66.67% (2/3)	33.33% (1/3)	66.67% (2/3)	33.33% (1/3)	66.67% (2/3)

PAX1 (hypo), PAX1 hypomethylation; PAX1 (hyper), PAX1 hypermethylation; JAM3 (hypo), JAM3 hypomethylation; JAM3 (hyper), JAM3 hypermethylation; “PAX1 (hyper)/JAM3 (hyper)” indicates either gene is hypermethylated; “PAX1 (hypo)/JAM3 (hypo)” indicates either gene is hypomethylated.”PAX1 (hyper) and JAM3 (hyper)” indicates both genes are hypermethylated; “PAX1 (hypo) and JAM3 (hypo)” indicates both genes are hypomethylated.

**TABLE 5:** Predictive performance of pretreatment PAX1 and JAM3 hypermethylation for PDT efficacy.

Methylation	PAX1 (hyper)	JAM3 (hyper)	PAX1 (hyper)/JAM3 (hyper)	PAX1 (hyper) and JAM3 (hyper)
CIN 2				
AUC	0.823	0.877	0.861	0.816
95% CI	0.732∼0.915	0.797∼0.957	0.807∼0.916	0.673∼0.959
*P*	< 0.001	<0.001	< 0.001	<0.001
Sensitivity	74.19%	80.65%	83.87%	67.74%
Specificity	90.46%	94.82%	88.28%	95.37%
CIN 3				
AUC	0.736	0.723	0.734	0.757
95% CI	0.530∼0.942	0.343∼1.000	0.6150∼0.8530	0.554∼0.960
*P*	0.025	0.028	0.008	0.022
Sensitivity	83.33%	100.00%	100.00%	83.33%
Specificity	63.83%	44.68%	53.19%	68.09%
VaIN 2				
AUC	0.871	0.655	0.777	0.655
95% CI	0.737∼1.000	0.428∼0.882	0.614∼0.940	0.428∼0.882
*P*	< 0.001	0.039	0.004	0.039
Sensitivity	58.33%	33.33%	66.67%	33.33%
Specificity	95.45%	97.73%	88.64%	97.73%
VaIN 3				
AUC	0.833	0.750	0.750	0.833
95% CI	0.401∼1.000	0.317∼1.000	0.317∼1.000	0.401∼1.000
*P*	0.048	0.102	0.102	0.048
Sensitivity	66.67%	66.67%	66.67%	66.67%
Specificity	100.00%	83.33%	83.33%	100.00%

## Discussion

Cervical cancer and its precursor lesions remain a substantial public health burden worldwide and in China (21, 22). In this prospective cohort study, we evaluated the therapeutic efficacy of PDT in patients with cervical and vaginal high-grade intraepithelial lesions and further explored the predictive value of pretreatment PAX1/JAM3 methylation status. Our results showed that PDT achieved favorable pathological remission rates, particularly in CIN2 and CIN3 lesions. More importantly, PAX1/JAM3 hypomethylation was associated with better treatment response, whereas hypermethylation was more frequently observed in patients with persistent disease. These findings suggest that pretreatment PAX1/JAM3 dual-gene methylation testing may help identify patients who are more likely to benefit from PDT and may provide a non-invasive tool for individualized treatment decision-making.

With the increased prevalence of cancer screening and advances in colposcopic technology, the detection rate of vaginal epithelial lesions has significantly improved. While the risk of progression from VaIN1 to invasive cancer is relatively low, VaIN2-3 carries an approximate 12% risk of malignant transformation (23), which increases to 16.7% in patients with a prior hysterectomy (24). Therefore, clinicians should prioritize the diagnosis and treatment of VaIN. In the present study, we included patients with VaIN2/3 and analyzed both treatment response and pretreatment methylation status in this subgroup. Although DNA methylation testing has been widely investigated in cervical lesions, data regarding its clinical value in vaginal intraepithelial lesions remain limited. Therefore, our findings may provide preliminary evidence for further studies on methylation-based risk stratification in VaIN.

Current standard treatments for HSIL remain predominantly destructive. Surgical resection may compromise cervical integrity and reproductive outcomes. Exploring minimally invasive treatments that preserve normal tissue structure while achieving comparable efficacy is of great clinical significance (25). Because CIN and VaIN are confined to the epithelial layer and the female lower genital tract is anatomically accessible for topical ALA application and light exposure, ALA-PDT is theoretically suitable for treating both cervical and vaginal intraepithelial lesions (26).

The efficacy of PDT for HSIL has been progressively validated with technological advancements. Previous studies have reported an effective rate of 86.3–92% for ALA-PDT in treating CIN2 (11, 27, 28); however, these studies were limited by small sample sizes, short follow-up durations, and strict inclusion criteria excluding patients with multiple comorbidities, post-resection recurrence, extensive lesions, or immune dysfunction. Compared with previous studies, the present cohort included a relatively large number of patients and provided additional prospective evidence regarding ALA-PDT for CIN2/3 and VaIN2/3. The remission rate for CIN2 was 92.21% (367/398), and notably, this study included CIN3 patients after thorough clinical evaluation and informed consent, with a remission rate of 88.68% (47/53). These findings provide additional prospective evidence supporting the potential clinical application of ALA-PDT in selected patients with CIN2/3, especially those who desire fertility preservation or are unsuitable for excisional treatment.

As a minimally invasive treatment for cervical and vaginal precancerous lesions, PDT efficacy may be associated with multiple factors. Previous research has shown that cytological abnormalities suggesting more severe lesions and lesion size are associated with ALA-PDT outcomes (29). In addition, larger cervical lesions may be associated with more severe disease or occult microinvasion (30). Therefore, comprehensive cytological, colposcopic, and pathological evaluation remains essential when selecting candidates for ALA-PDT. For patients with extensive CIN3 lesions, close post-treatment monitoring and timely discussion of alternative therapies are necessary if the response is unsatisfactory.

The depth of VaIN lesions ranges from 0.1 to 1.4 mm, and 635 nm wavelength light can penetrate > 2 mm, allowing ALA to reach the entire vaginal epithelium. Our team previously published data on the efficacy and influencing factors of ALA-PDT for VaIN (13), with results consistent with the present study. Key factors affecting treatment outcomes include drug penetration/absorption and adequate exposure of lesions, suggesting that patient age and prior hysterectomy may modulate ALA-PDT efficacy.

With the expanding application of PDT, identifying patients most likely to benefit from this treatment is critical. However, the lack of effective preoperative predictive markers currently results in suboptimal outcomes for some individuals.

From an epigenetic perspective, aberrant host-cell DNA methylation is closely involved in carcinogenesis and the progression of cervical precancerous lesions (31–34). PAX1 and JAM3 methylation assays have been widely investigated as molecular tools for cervical lesion screening, triage, and risk stratification (15–18, 35–38). In contrast to conventional cytology or HPV testing, methylation testing reflects host-cell molecular alterations and may provide additional information regarding lesion biology. Previous studies have shown that PAX1 methylation is frequently detected in CIN3 and invasive cervical cancer (39, 40), and experimental or clinical evidence has suggested that methylation-related gene regulation may be associated with treatment sensitivity in cervical cancer (41, 42).

Although most previous studies have focused on the diagnostic or prognostic value of methylation testing, limited evidence suggests that methylation status may also be associated with ALA-PDT response. Tang et al. reported that patients with PAX1 hypomethylation had higher HPV clearance and complete response rates after ALA-PDT than those with PAX1 hypermethylation (43). Consistent with this finding, our study showed that patients with PAX1/JAM3 hypomethylation had higher remission rates, whereas hypermethylation was more common among patients with persistent disease. These findings support the potential role of pretreatment methylation testing as a predictive biomarker for pathological response after ALA-PDT.

In the present cohort, the proportion of PAX1/JAM3 hypomethylation was relatively high, particularly among patients with CIN2. This distribution differs from some previous studies reporting higher methylation rates in CIN2/3 populations. Several factors may partly explain this discrepancy, including differences in sample collection methods, population characteristics, lesion composition, and the fact that our cohort consisted of patients selected for ALA-PDT rather than an unselected HSIL screening population. Moreover, this study did not include normal cervical controls, and baseline methylation distributions across the full spectrum of normal cervix, LSIL, HSIL, and invasive cancer were not evaluated. Further studies including normal controls and broader disease-grade comparisons are needed.

Combined detection of PAX1 and JAM3 methylation may improve prediction compared with single-gene testing, as multi-gene panels can better reflect molecular heterogeneity within lesions. In the present study, combined methylation testing showed potential value for predicting incomplete response to ALA-PDT. For patients with pretreatment hypermethylation, closer follow-up and timely reassessment after ALA-PDT may be warranted. Conversely, patients with hypomethylation may be more likely to benefit from ALA-PDT and may represent suitable candidates for this tissue-sparing treatment. In addition, the use of exfoliated cell samples is non-invasive, convenient, and clinically feasible.

However, this study has several limitations: (1) although the overall sample size was relatively large, the numbers of patients with CIN3 and especially VaIN3 were limited, which may reduce the stability of subgroup analyses and ROC estimates; (2) this was a single-center prospective cohort study, and external validation is needed; (3) only two methylation markers, PAX1 and JAM3, were evaluated, and additional biomarkers or multi-omics approaches may further improve predictive accuracy; and (4) the molecular mechanisms underlying the association between methylation status and ALA-PDT response were not experimentally investigated in this study. Therefore, mechanistic interpretations should be considered hypothetical, and further basic research is needed.

## Conclusion

ALA-PDT is an effective treatment for HSILs, with demonstrated efficacy in HR-HPV clearance. Patients with PAX1 and JAM3 hypomethylation achieved higher remission rates compared to those with hypermethylation. Combined detection of PAX1 and JAM3 methylation status in exfoliated cells provides a potentially useful non-invasive approach for predicting pathological response to ALA-PDT, offering a scientific basis for individualized clinical decision-making. Future research should focus on optimizing the detection system, refining prediction models, including normal controls and external validation cohorts, and promoting widespread clinical application to improve outcomes for patients with cervical and vaginal precancerous lesions.

## Data Availability

The raw data supporting the conclusions of this article will be made available by the authors, without undue reservation.
