# Supplementary material for: Efficacy of 5-aminolevulinic acid photodynamic therapy for the treatment of female lower reproductive tract intraepithelial lesions and its predictive biomarker: DNA methylation
Source: Front Med (Lausanne). 2026 Jun 25;13:1828763. doi: 10.3389/fmed.2026.1828763 (PMC13346238; doi:10.3389/fmed.2026.1828763)
Supplement: Supplementary file 1 [file Supplementary_file_1.docx]

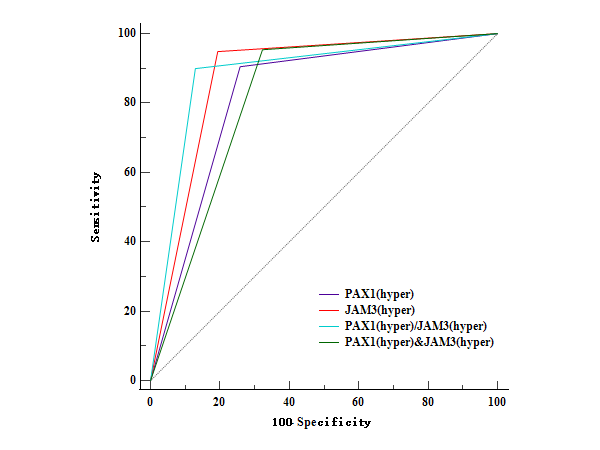

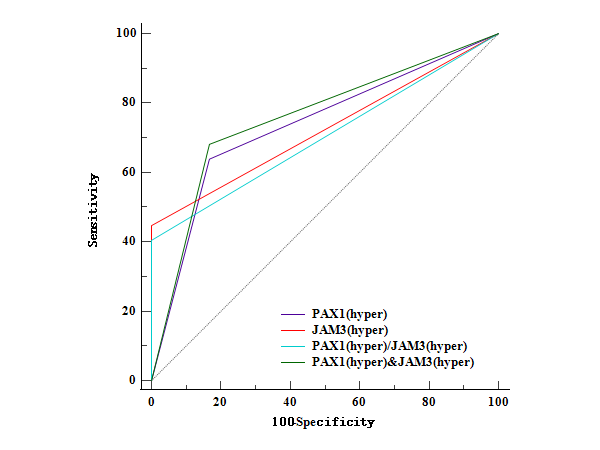


A B


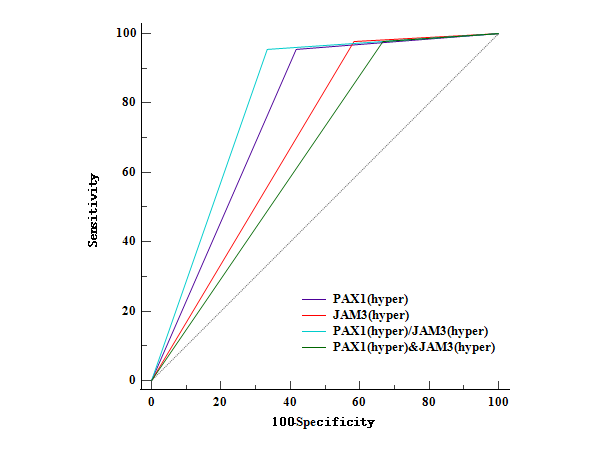

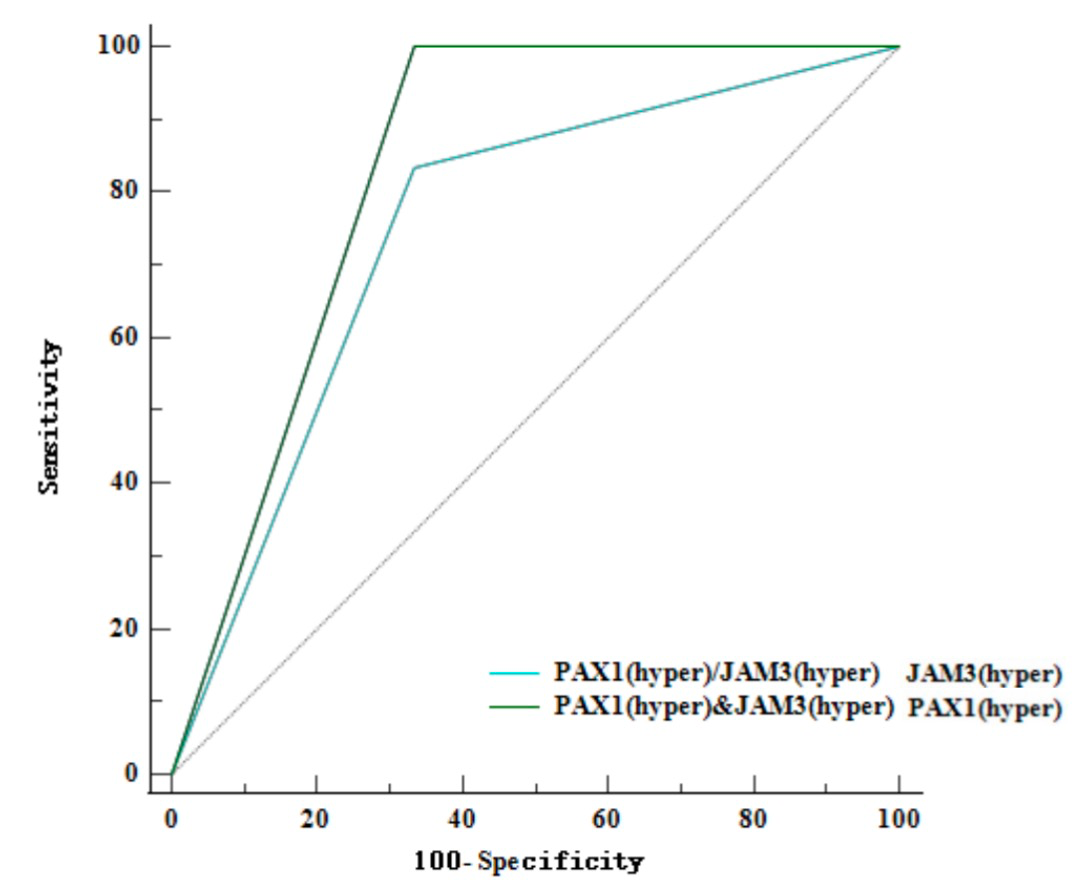


C D

Supplementary Figure 1. Receiver operating characteristic (ROC) curves of PAX1(hyper), JAM3(hyper), PAX1(hyper)/JAM3(hyper) and PAX1(hyper)&JAM3(hyper) in predicting theraputic response. CIN 2 (A), CIN 3 (B), VaIN 2 (C) and VaIN 3 (D).
